# Biologically‐oriented alveolar ridge preservation to correct bone dehiscence at immediate implant placement

**DOI:** 10.1002/cap.10334

**Published:** 2025-01-15

**Authors:** Leonardo Trombelli, Tommaso Grenzi

**Affiliations:** ^1^ Department of Periodontal and Peri‐implant Diseases University of Ferrara Ferrara Italy; ^2^ Operative Unit of Dentistry Azienda Unità Sanitaria Locale Ferrara Italy

**Keywords:** alveolar ridge preservation, guided bone regeneration, immediate implant placement, peri‐implant bone dehiscence

## Abstract

**Background:**

The purpose of the present case study is to describe the application of a modification of the Biologically‐oriented Alveolar Ridge Preservation (BARP) principles in cases of peri‐implant bone dehiscence (PIBD) due to a compromised alveolus at immediate implant placement (IIP).

**Methods:**

The technique is based on the stratification of three layers: a deep layer with a collagen sponge (CS) in the apical part of the alveolus (where the buccal bone plate was still present) to support the blood clot; a graft layer to correct the PIBD; and a superficial collagen layer to cover the graft thus providing space and enhancing clot/graft stability. Healing was obtained by primary closure.

**Results:**

At the re‐entry procedure for implant uncovering, a complete PIBD correction with newly formed peri‐implant bone up to the level of the polished collar was observed in both cases.

**Conclusions:**

These observations suggest that BARP based on the combined use of CS and deproteinized bovine bone mineral may be regarded as a simplified treatment option to correct a PIBD at IIP.

**Key points:**

- Why treat a Peri‐Implant Bone Dehiscence (PIBD)? PIBD should be treated to avoid biological and esthetic complications over time.
- What plays a key role in this case? The stability of both the graft and the cloth is essential for providing space for bone formation to correct the PIBD; the extraction socket supports angiogenic and osteogenic properties; Primary intention closure is crucial to prevent potential infection.
- Limitation: the efficacy of the technique must be assessed.

**Plain language summary:**

This case study described the potential to correct a post‐extraction osseous defect associated with a substantial portion of a dental implant which resulted exposed and without bone support on its buccal aspect. The application of a novel bone augmentation technique, namely the biologically oriented Alveolar Ridge Preservation, has been described. This simplified procedure is based on the stratification of i) a deep collagen layer in the apical part of the socket to support the blood clot and spontaneous bone formation, ii) a graft of bone substitute to correct the missing bone, and iii) a superficial collagen layer to protect the graft and the wound. After 5 months, a complete correction of the osseous defect with newly formed bone up to the head of the implant was observed in both treated cases.

## INTRODUCTION

Immediate implant placement (IIP) is a surgical procedure that offers several advantages: reduced chair time, lower costs, and greater comfort for both the patient and the operator.^1^ On the other hand, the major challenge of this treatment lies in the management of the post‐extraction socket to create favorable healing conditions for the peri‐implant soft and hard tissues.^2^, ^3^ This management adds to the complexity of the technique and requires significant operative experience. Although an ideal pre‐requisite to perform an IIP is a post‐extraction site with an intact buccal wall, endodontic/periodontal infection combined with specific anatomy of the alveolar bone along with the tooth position may often set conditions for an increased incidence of buccal bone dehiscence at tooth extraction.^4^, ^5^ In such cases, the reconstruction of the post‐extraction socket is usually based on a Guided Bone Regeneration (GBR) procedure aimed at restoring the missing alveolar walls thus limiting the incidence of biological and aesthetic complications over time.^6^, ^7^, ^8^ The regenerative approach includes the use of bone grafts/substitutes, barrier membranes, bioactive factors, and cell therapies.^9^ A combination of non‐resorbable/resorbable membrane and graft biomaterial is the most common treatment option in clinical practice.^9^, ^10^

Recently, Pramstraller et al.^11^ have proposed a novel technique for alveolar ridge preservation (ARP), namely the Biologically‐oriented Alveolar Ridge Preservation (BARP), consisting of the stratification of a collagen sponge (CS) and a particulate xenograft. BARP has been shown effective for maintaining the ridge dimensions and limiting the need for additional bone augmentation at implant placement in case of both intact and compromised alveoli.^12^ The rationale of BARP is based on 1) optimized conditions for wound maturation in the middle and apical parts of the socket by avoiding any potential interference of the bone graft with spontaneous bone deposition^13^, ^14^; 2) provision of ARP by grafting only the coronal portion (≈4–5 mm) of the socket to effectively mitigate bone remodeling where most clinically relevant^13^, ^15^, ^16^; and 3) enhanced provision for clot and graft stabilization at socket entrance. Histologically, the deep collagen layer appeared to effectively support the clot during the bone healing process. In the apical and central parts of the socket, abundant trabeculae of mature lamellar bone were evident, with minor signs of bone remodeling and minimal inflammation.^12^ Moreover, the deep collagen layer was able to maintain the graft particles mostly confined to the coronal portion of the socket throughout the tissue maturation phase. The superficial layer of collagen sponge used to stabilize the clot and graft at the socket entrance allowed for a successful re‐epithelialization of the area left intentionally exposed.^11^, ^17^

In our previous study,^12^ BARP has also shown effective in cases where the position of the buccal and/or lingual bone wall was at least 3 mm more apical with respect to the line passing through the adjacent interdental bone septa. In such cases, an additional collagen sponge was interposed between the residual cortical bone plate and the mucoperiosteal flap to create a containing envelope. The purpose of the present case study was to preliminary evaluate whether and to what extent a surgical technique based on BARP principles and procedure may be used to correct a compromised alveolar bone wall associated with a PIBD at IIP.

## MATERIALS AND METHODS

### Case management

The present Case Study was prepared in accordance with the Case Report (CARE) guidelines.^18^

Two patients, both affected by controlled (HbA1c < 6%) type 2 diabetes mellitus (T2DM), non‐smokers, not taking drugs that could interfere with bone metabolism, and showing plaque and bleeding scores ≤ 25%, were selected for treatment. Both patients were treated at a private clinic by an expert periodontist (LT).

Patients received premedication with 2 g of amoxicillin and clavulanic acid 1 h before surgery. After signing a written informed consent for the treatment, preoperative local anesthesia was administered using articaine with 1:100,000 vasoconstrictor.* During surgery, a buccal full‐thickness flap was elevated and the tooth was extracted as delicately as possible. The implant site was then prepared with drills following the manufacturer's instructions. Tissue‐level implants† were equi‐crestally positioned with the 1 mm polished collar placed at the level of the palatal bone plate. The BARP procedure was performed as follows:

‐ A trimmed portion of a CS‡ was placed in the apical part of the alveolus (where the buccal bone plate was still present) to support the blood clot;
‐ A particulate deproteinized bovine bone mineral (DBBM)§ combined with either autologous bone particles or a polynucleotide‐based (PN) + hyaluronic acid (HA) healing accelerator (PN‐HA) gel¶ was placed to correct the PIBD up to the level of the polished collar;
‐ An additional trimmed CS was placed over the graft and extended to cover the occlusal portion of the implant in order to provide space and enhance clot/graft stability.

Healing was obtained with the primary intention of using 5/0 and 6/0 absorbable sutures.# Patients were prescribed Ibuprofen 600 mg pro re nata and chlorhexidine mouthwash, 10 mL t.i.d for 21 days. Sutures were removed at 14 days post‐surgery.

### Case 1

**FIGURE 1:**
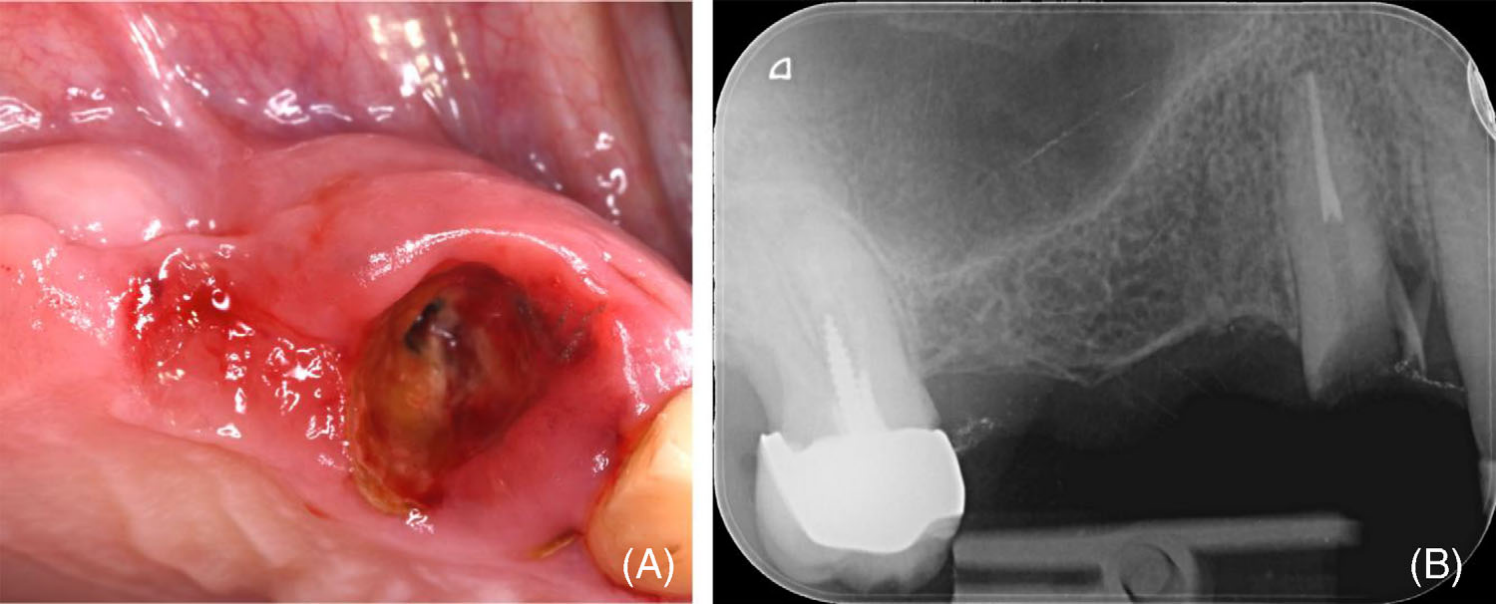
(A) Clinical image of the preoperative situation showing a root fracture on upper right cuspid (tooth #6); (B) preoperative x‐ray.

**FIGURE 2:**
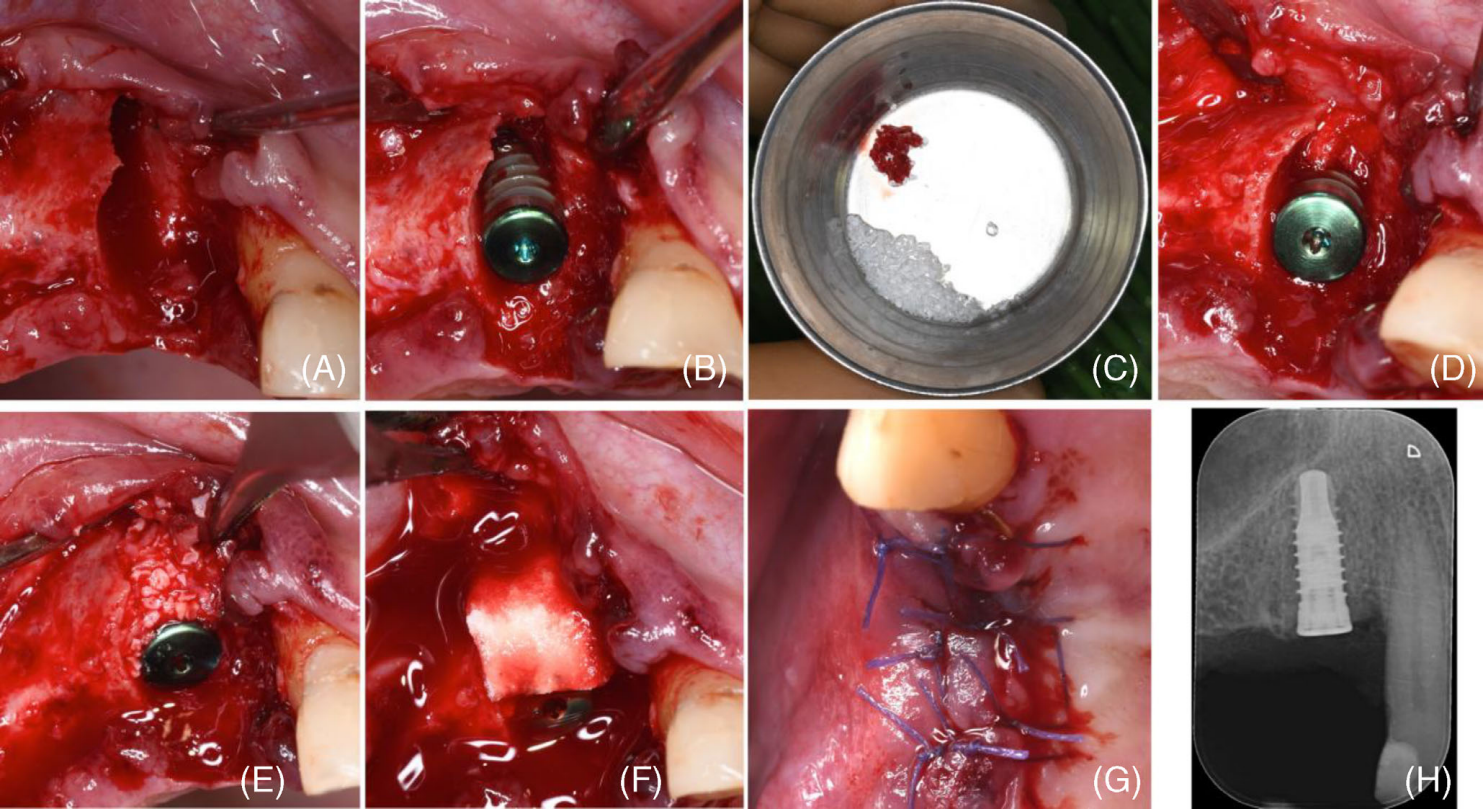
(A) Post‐extraction site with 6 mm deep buccal bone dehiscence; (B) A 5 mm wide and deep, U‐shaped peri‐implant bone dehiscence (PIBD) is evident at implant placement; (C) graft composition: autologous cortical bone particles (ACBP) scraped from the same surgical site and deproteinized bovine bone mineral (DBBM); (D) a trimmed collagen sponge (CS) placed in the apical part of the alveolus; (E) ACBP were partly placed in direct contact with the exposed portion of the implant, partly mixed with the xenograft to overcorrect the PIBD; (F) a trimmed CS placed onto the graft in order to stabilize it and provide space; (G) primary intention wound closure with 5/0 absorbable suture; (H) postoperative x‐ray.

The patient, a male, 67 years old showed a root fracture associated with an infra‐bony defect on the upper right cuspid (tooth #6) (Figure 1A,B) on November 2023. After tooth extraction and careful debridement of the alveolus, a compromised buccal bone wall up to the apical third of the root was evident (Figure 2A). After implant positioning (4 mm diameter and 11 mm length; SPI Inicell Element†) a PIBD 5 mm wide and 5 mm deep was present on the buccal aspect (Figure 2B). A combination of autologous cortical bone particles (ACBP), harvested with a bone scraper∥ from the distal edentulous ridge, and DBBM was used to correct the PIBD (Figure 2C). A trimmed CS was placed in the most apical portion of the alveolus (Figure 2D). ACBP were partly placed in direct contact with the exposed portion of the implant and partly mixed with the xenograft to overcorrect the defect (Figure 2E). The graft was then stabilized by a second collagen layer which was extended over the cover screw (Figure 2F). The flap was coronally advanced and closed with an internal mattress and interrupted sutures to favor primary intention healing (Figure 2G,H).

### Case 2

**FIGURE 3:**
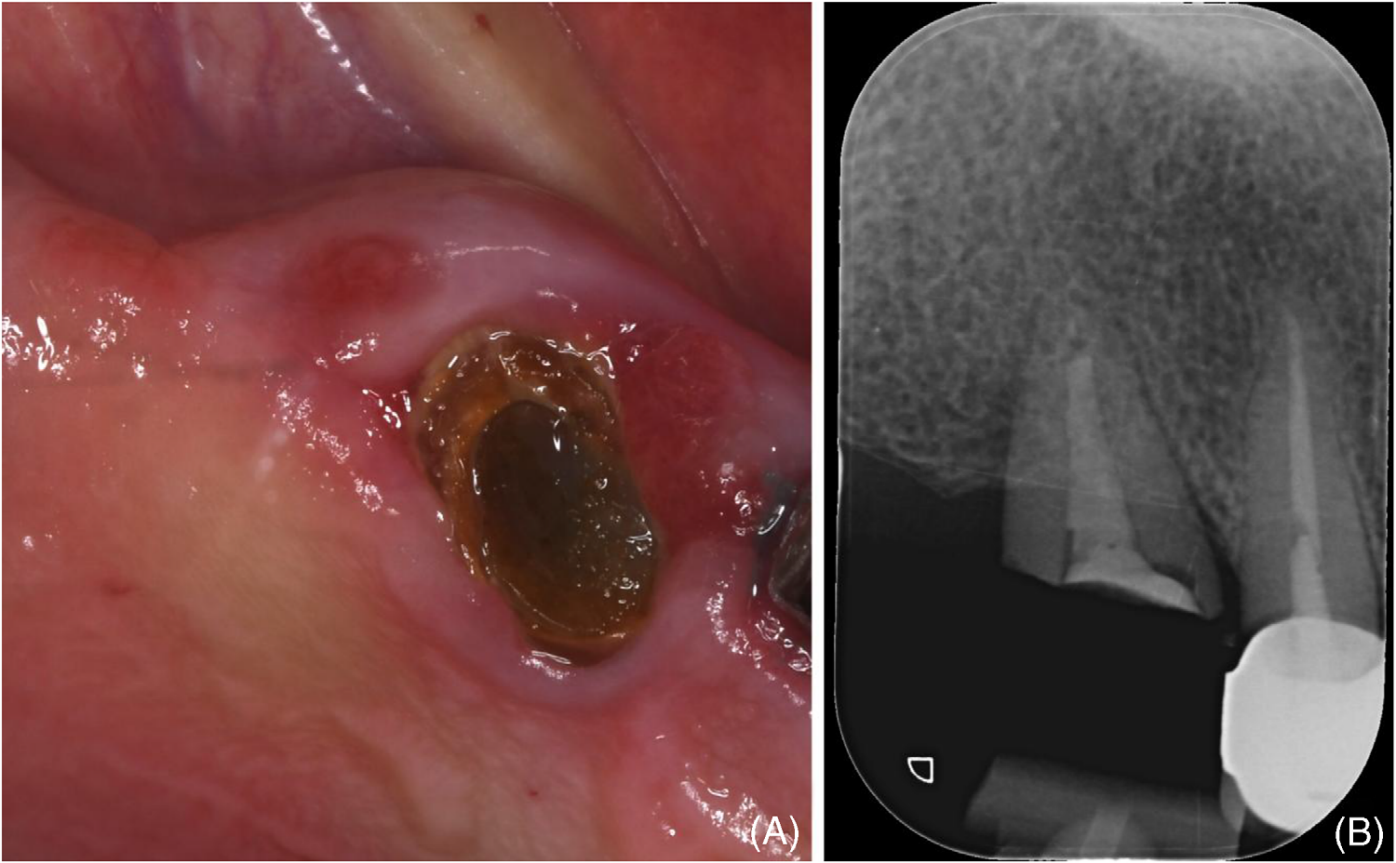
(A) Root fracture on on upper right cuspid (tooth #6); (B) preoperative x‐ray.

**FIGURE 4:**
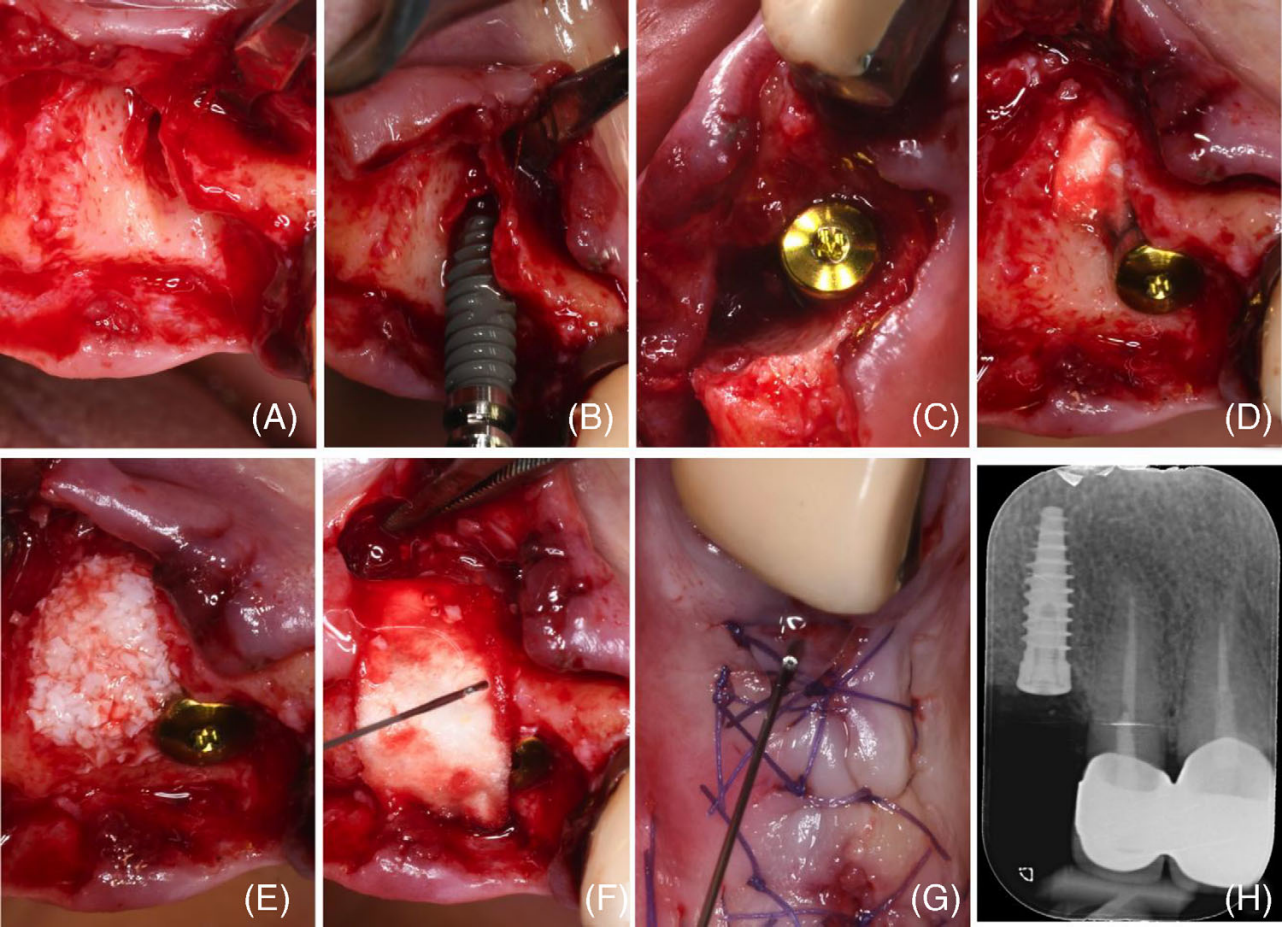
(A) Root extraction led to a severe buccal, V‐shaped bone dehiscence; (B) implant insertion resulting in a 5‐mm peri‐implant bone dehiscence (PIBD); (C) occlusal view of the buccal bone defect; (D) a trimmed collagen sponge (CS) was placed in the most apical part of the alveolus; (E) PIBD correction with deproteinized bovine bone mineral (DBBM) blended with HA‐PN gel; (F) a trimmed CS placed over the graft and soaked with PN‐HA gel; (G) primary intention closure and PN‐HA gel application; (H) postoperative x‐ray.

The patient, a female, 72 years old, presented a root fracture on the upper right cuspid (tooth #6) (Figure 3A,B) associated with a buccal bone dehiscence involving two‐thirds of the root length (Figure 4A) in February 2024. Implant placement (3.5 mm diameter and 11 mm length; NEVO Implant†) resulted in a PIBD 4 mm wide and 5 mm deep at the distobuccal aspect (Figure 4B,C).

A trimmed CS was placed in the most apical portion of the alveolus (Figure 4D). The bone defect was corrected by DBBM mixed with a PN‐HA gel (Figure 4E). The graft was then stabilized by a second CS layer extended onto the graft and soaked with the PN‐HA gel (Figure 4F). Primary closure was achieved by a coronally advanced flap fixed with an internal mattress and interrupted sutures. The wound was conditioned by an extra application of PN‐HA gel (Figure 4G,H).

## RESULTS

**FIGURE 5:**
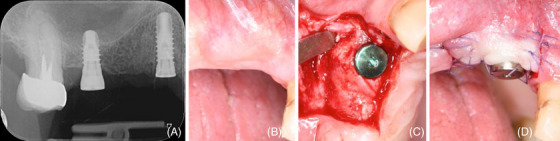
(A) Preoperative X‐ray taken before 2nd stage surgery; (B) clinical image before 2nd stage surgery; (C) clinical image showing the peri‐implant bone thickness and complete peri‐implant bone dehiscence (PIBD) correction; (D) APF sutured with 6/0 absorbable suture.

**FIGURE 6:**
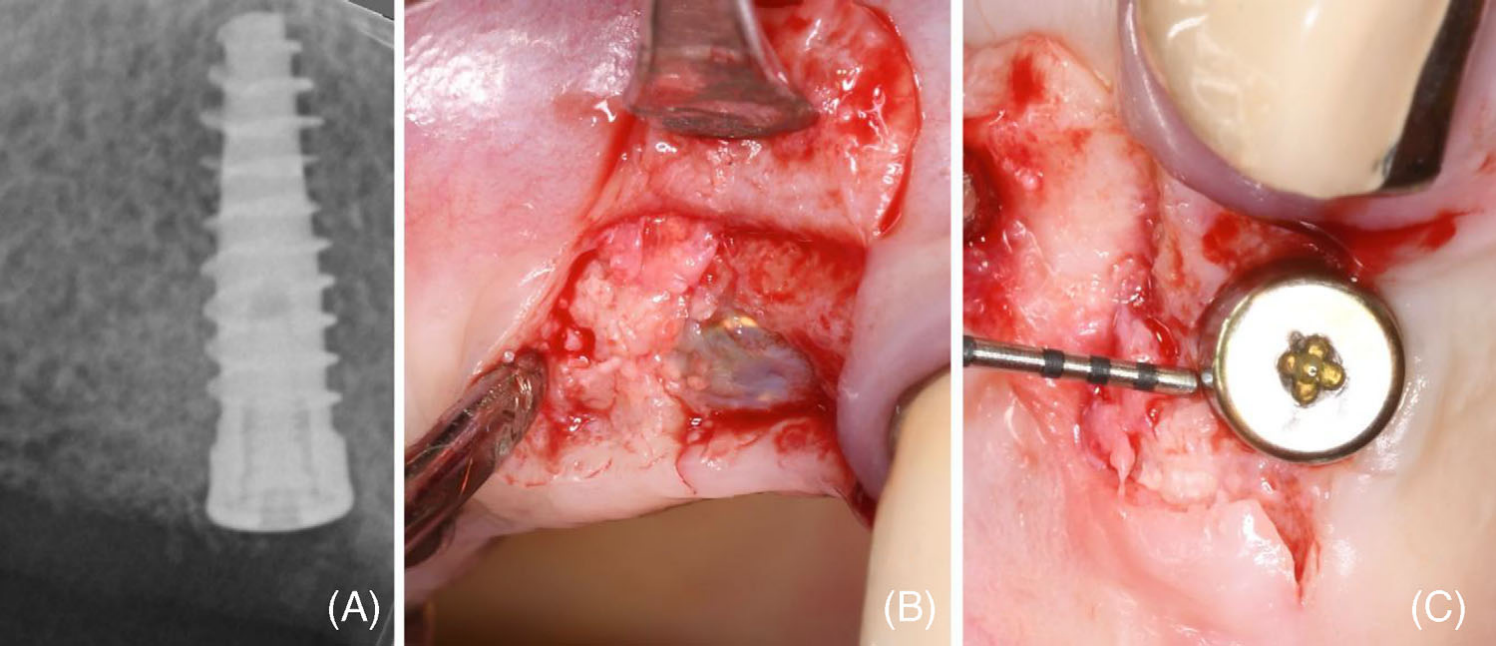
(A) Radiographic aspect at 5 months; (B) at re‐entry, a completely corrected peri‐implant bone dehiscence (PIBD) was evident; (C) a 1 mm newly formed bone was present up to the polished implant collar.

Healing was uneventful and implant uncovering was performed at 5 months post‐surgery by a buccal full‐thickness flap that was apically positioned to increase the height and thickness of the buccal keratinized mucosa both in Case 1 (Figure 5A,B,D) and in Case 2 (Figure 6A,B).

**FIGURE 7:**
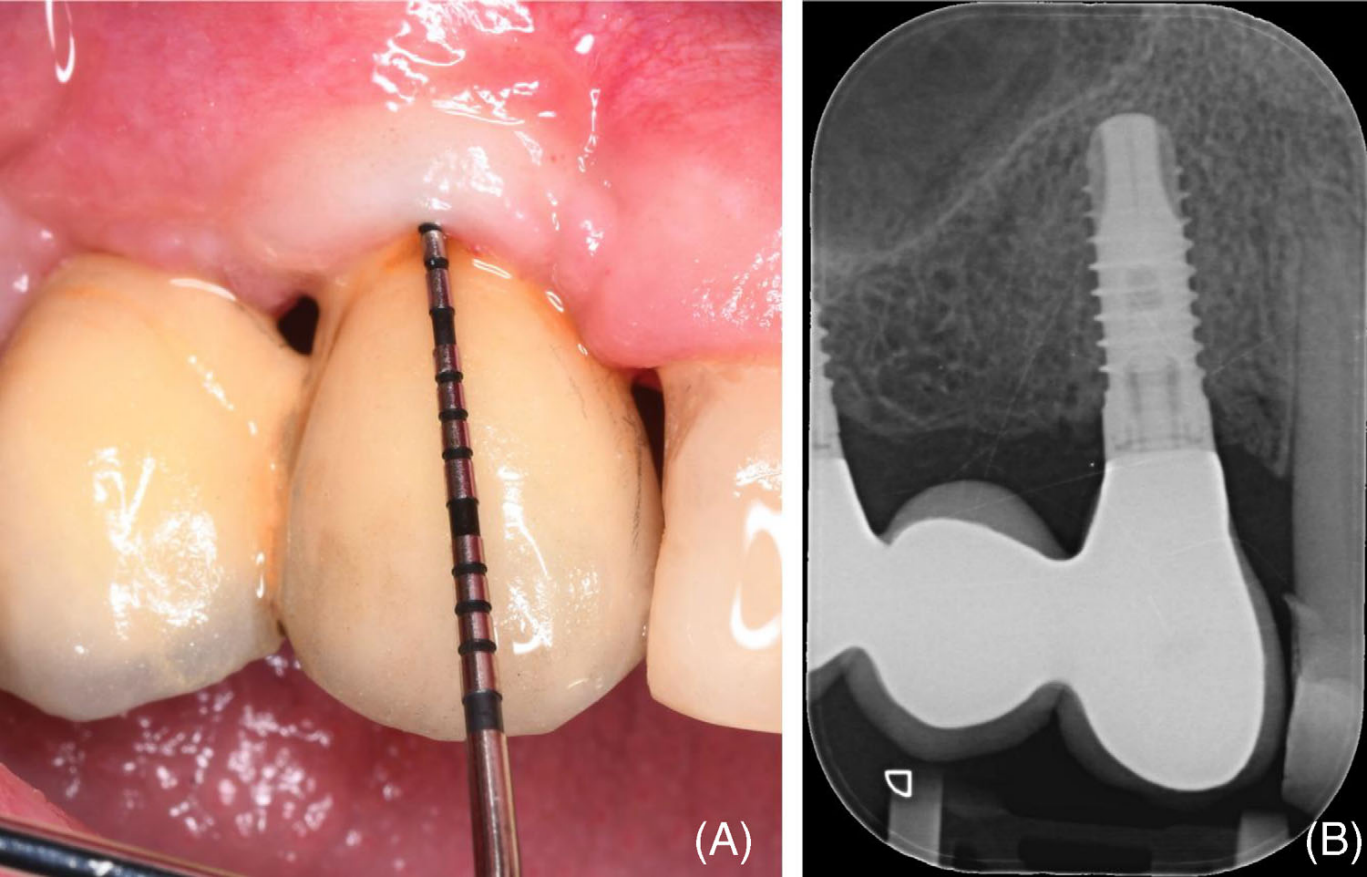
(A) Final prosthetic restoration; (B) radiographic view at prosthetic loading.

**FIGURE 8:**
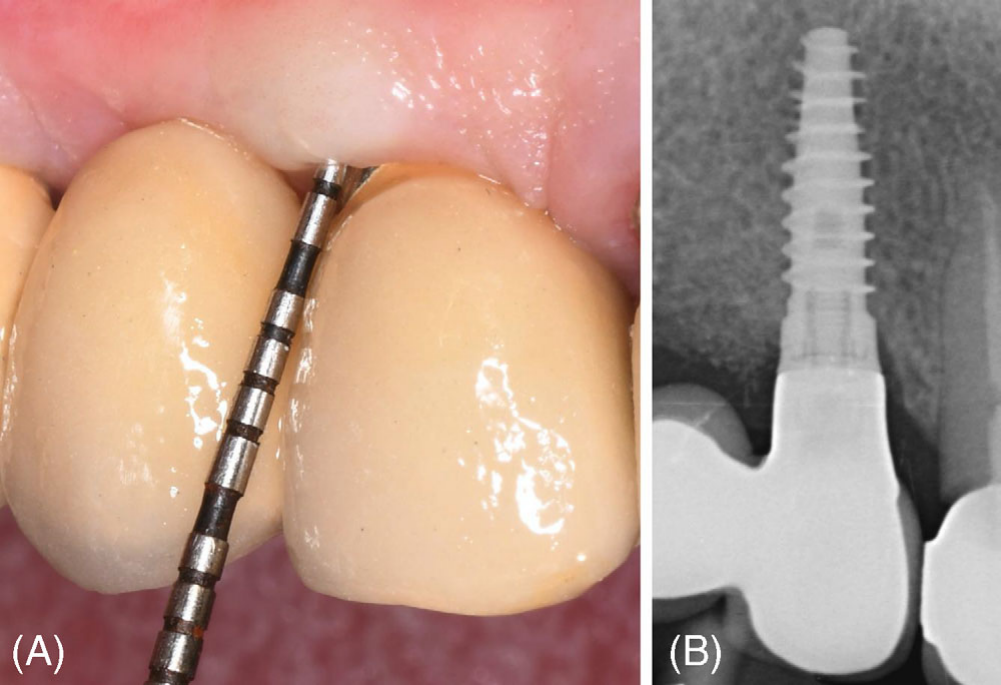
(A) Final prosthetic restoration; (B) radiographic view at prosthetic loading.

A complete PIBD correction with newly formed peri‐implant bone up to the level of the polished collar was observed in both Case 1 (Figure 5C) and Case 2 (Figure 6C).^19^ Figures 7A,B and 8A,B show the clinical and radiographic aspects at the time of prosthetic restorations.

## DISCUSSION

Previous studies have demonstrated that BARP may represent an effective approach to maintaining the bone volumes following tooth extraction at both intact and compromised alveoli.^11^, ^12^ The present case study seems to indicate that this technique may also be a suitable option to treat a compromised alveolus associated with a PIBD. In both cases, the procedure resulted in a complete PIBD correction at the level of the polished collar at 5‐month re‐entry.

In our study, both patients were affected by controlled T2DM. While most of the clinical studies evaluating bone augmentation procedures exclude patients with DM, previous studies have shown a successful and uncomplicated treatment outcome following augmentation techniques in DM patients.^20^, ^21^ Since data regarding the success rate of alveolar bone augmentation in DM are still inconclusive due to scarce and extremely heterogeneous evidence, the reliability of any bone augmentation procedure when applied to DM patient needs to be thoroughly assessed.^22^

In 2006, Wang e Boyapati described the PASS principles for bone regeneration^23^ encompassing primary closure, angiogenesis, space provision, and wound stability. In our cases, the deep collagen layer may have optimized conditions for wound stability leading to spontaneous bone deposition in the apical part of the socket. Space provision at PIBD was mainly achieved by the use of DBBM which has been repeatedly demonstrated to be effective as an osteoconductive scaffold to support new bone formation in case of compromised alveoli with^24^, ^25^ and without IIP.^12^, ^26^ Moreover, a second CS layer has been used to stabilize the graft while allowing the angiogenic and osteogenic properties of the periosteum from the mucoperiosteal flap to contribute to new bone formation.^27^ Primary intention closure was provided to enhance an undisturbed wound healing process.

Due to the severity of the bone defect, a combination of ACBP and DBBM was used in Case 1. The use of such composite bone graft either in two layers^28^, ^29^ or mixed^30^, ^31^, ^32^ has been repeatedly shown effective in various bone augmentation procedures. The rationale is based on the observed beneficial effect of adjunctive cancellous bone chips to DBBM alone when used for a GBR procedure in vitro and in vivo.^33^

In Case 2, defect resolution was achieved by the BARP procedure combined with DBBM and PN‐HA gel. The assumption is that the adjunctive effect of this healing accelerator may have both enhanced DBBM osteoconductive properties and facilitated wound closure. In a previous study, the additional use of a cross‐linked, high molecular weight HA (xHyA) to DBBM used for GBR showed a statistically higher crestal ridge dimension compared to DBBM alone. Histologically, a tendency for greater mineralized tissue formation for xHyA+DBBM compared to DBBM was observed which contained a higher amount of new bone and less DBBM residues.^34^ A faster bone formation was reported in cortical tibia defects when deproteinized porcine bone granules were impregnated with PN gel compared to biomaterial alone.^35^ Recently, a combined PN‐HA gel was shown safe and effective in promoting bone formation in localized horizontal bone augmentation procedures prior to implant placement when used in conjunction with DBBM and collagen membrane.^36^ The additional PN component to HA gel may have added a beneficial effect on soft tissue healing and wound closure. In this respect, PNs have been shown to accelerate wound healing compared to HA alone in vivo.^37^, ^38^

A superficial collagen layer was used to stabilize the graft and enhance blood clot stability. The use of this device is based on the idea that wound stability and space provision may be more relevant than cell exclusion to favor bone regeneration. In this respect, a randomized clinical trial has demonstrated that CS is similarly effective to a collagen membrane when used with Freeze‐Dried Bone Allograft (FDBA) to preserve ridge dimension compared to spontaneous healing.^17^ Moreover, CS seems to block soft tissue infiltration and guarantee graft stability when used for ARP procedures.^39^

Although it may be considered a proof‐of‐principle to show the potential of the BARP technique to completely correct a PIBD at IIP, this case study suffers from limitations, including the lack of heterogeneity in case selection and a standardized regenerative approach based on patient‐ and defect‐characteristics. Both treated cases refer to upper canines showing a clinically relevant PIBD at tooth extraction. Our intention was to explore the effect of the procedure in an esthetically sensitive clinical scenario where tooth extraction may be frequently hampered by a thin/compromised buccal alveolar wall. Further evaluations based on a structured study design are mandatory to assess whether and to what extent the BARP procedure may compare to any available documented treatment options when used in different clinical conditions. Also, the stability of the augmented bone and peri‐implant healthy condition needs to be determined by long‐term radiographic and clinical assessment.

## CONCLUSIONS

In conclusion, the present case study suggests that under specific circumstances BARP based on the combined use of CS and DBBM may be regarded as a simplified treatment option to correct a PIBD at IIP.

## AUTHOR CONTRIBUTIONS

Prof. Leonardo Trombelli conceptualized the idea, managed the surgery, and reviewed the manuscript. Dr. Tommaso Grenzi collected data and wrote the original draft.

## CONFLICT OF INTEREST STATEMENT

The authors declare no conflicts of interest.

## Data Availability

Data sharing is not applicable to this article as no datasets were generated or analyzed during the current study. All significant clinical photographs are provided in the *Figures* section. These images document the clinical procedures involved, including tooth extraction, compromised alveolus, implant placement, and the bone regeneration process. Furthermore, the extent of newly formed bone is clearly illustrated in the uploaded figures.

## Footnotes

* Optocain, with adrenaline 20mg/mL + 10µg/mL, AIC Molteni Dental srl

† Thommen Medical AG, Grenchen, Switzerland

‡ Collafleece, Botiss biomaterials GmbH, Zossen, Germany

§ Bio‐Oss, Geistlich Pharma AG, Wolhusen, Switzerland

¶ Regenfast; Officina Biofarmaceutica Mastelli Srl, Sanremo, Italy

# Vycril, Ethicon, Somerville, New Jersey

∥ SafeScraper TWIST, Geistlich Pharma AG, Wolhusen, Switzerland
